# Ten-Year Results of the Fitmore^®^ Hip Stem with a Focus on Varus/Valgus Alignment and Subsidence—A Retrospective Monocentric Analysis

**DOI:** 10.3390/jcm13185570

**Published:** 2024-09-20

**Authors:** Ronald Wanner, Christopher Butler Ransohoff, Tobias Wyss, Hubert Nötzli

**Affiliations:** 1 Department of Orthopaedics, Emmental Hospital, 3400 Burgdorf, Switzerland; 2 Department of Orthopaedics and Trauma Surgery, Inselspital, 3010 Bern, Switzerland; christopher.butler@insel.ch; 3 Orthopädie Sonnenhof, 3006 Bern, Switzerlandhubertnoetzli@sonnenhof.ch (H.N.); 4 Medical School, University of Bern, 3008 Bern, Switzerland

**Keywords:** hip replacement, total hip arthroplasty, minimally invasive surgery, long-term outcome, short stem, subsidence

## Abstract

**Background:** Hip arthroplasty is a common elective surgery worldwide, with rising numbers due to demographic changes and an emphasis on maintaining physical activity in the elderly. The development of new implant designs, especially shorter uncemented stems, has contributed to the advancement of minimally invasive implantation techniques. However, the long-term in vivo behaviour of these implants, particularly regarding subsidence, stability, and stress shielding, remains to be fully understood. **Methods:** This retrospective, monocentric cohort study analyses the long-term radiographic outcomes of the first 141 patients who underwent total hip arthroplasty with the Fitmore^®^ Hip Stem between June 2007 and December 2008. It focuses on subsidence, stability, varus–valgus alignment, and the influence of patient-related, anatomical, and surgical factors on implant behaviour over a 10-year follow-up period. **Results:** The average change in varus/valgus alignment was 0.7° into varus and the average subsidence was 1.7 mm over 10 years, with most changes occurring within the first six weeks postoperatively. The varus–valgus alignment and subsidence did not significantly change after the first year, indicating stable osteointegration of the implant. Neither patient factors (gender, age) nor surgical and implant factors (implantation angle, approach, stem family, size, total offset) had a significant influence on the long-term behaviour of the implant. **Conclusions:** The Fitmore^®^ Hip Stem shows highly reliable long-term stability and integration, unaffected by various patient, surgical, and implant factors, as confirmed by excellent register data. Nevertheless, monitoring of this and other new implants should be continued in order to determine implant behaviour, possible weaknesses, and indication limits at an early stage for the benefit of the patient.

## 1. Introduction

Hip arthroplasty is one of the most often performed elective operations worldwide and in Switzerland as well. According to the Swiss Implant Register (SIRIS), a total of over 24,000 hip arthroplasties were performed in Switzerland in 2021, of which almost 22,000 were primary total hip arthroplasties (THA) [1]. These numbers have been continuously rising since the start of recording in Switzerland. The reasons for the steadily rising number of THAs can be found in the demographic development of the country, which is marked by continuous population growth, an aging population and steadily increasing life expectancy [2]. Furthermore, recent findings in the field of gerontology have emphasised the importance of a sustained level of physical activity in the elderly to reduce morbidity and delay the development of frailty, which in turn requires functioning joints [3,4,5,6]. Due to its unrivalled success with satisfaction rates of well over 90%, the procedure of total hip arthroplasty was awarded the unofficial title of “Operation of the Century” in 2007 [7,8].

In addition to medical considerations, there are significant economic interests in this market, which have led to substantial investment in the development and refinement of new implants, a trend that continues to this day. Over the past few decades, there has been an unbroken trend towards uncemented short stems [9]. Uncemented stems that utilise the anatomical pathway potentially confer several advantages. These stems primarily anchor proximally, facilitating the introduction of load into the femoral metaphysis. This mode of anchoring may decrease stress shielding and cortical atrophy, thereby possibly creating more favourable conditions for subsequent revision surgeries. Furthermore, the design of short, curved stems enables the use of minimally invasive implantation techniques, which have superseded older methods involving muscle transection and detachment. According to the SIRIS report, over 80% of all primary total hip arthroplasties in Switzerland between 2016–2021 were performed via direct anterior or anterolateral approaches, both so-called minimally invasive [1].

**Figure 1:**
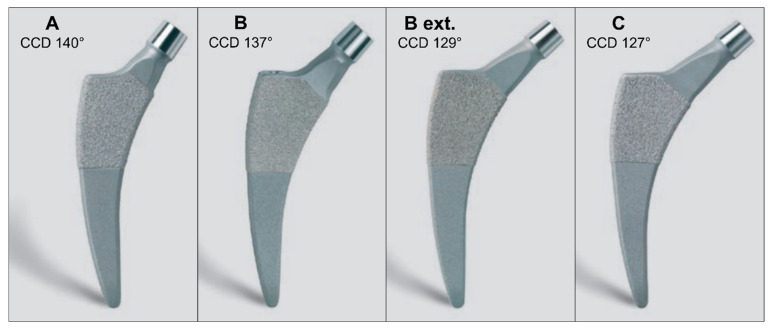
The four different stem families with a different medial curvature for families (**A**,**B**) (including (**B extended**) offset) and (**C**) resulting in different CCD angles. Each family consists of 14 stem sizes, with the CCD angle and neck length remaining unchanged within each family as the stem body grows sideways. CCD: Caput-Collum-Diaphyseal.

The Fitmore^®^ Hip Stem (Zimmer Biomet, Warsaw, IN, USA) is an example of this development and has played a pioneering role since its first implantation in 2007. The stem is a B3 short stem with single modularity according to the Radaelli classification made of a titanium alloy, is curved, triple-tapered, and has a trapezoidal cross-section [10]. Proximally, it is coated with a sprayed Ti-VPS coating (Titanium Vacuum Plasma Spray), and distally, it is rough blasted [11,12,13]. The osseous anchorage is primarily metaphyseal. The design allows for implantation along anatomical structures, which specifically spares the greater trochanter and the muscles attached to it. The Implant is suitable for all approaches, including trochanteric osteotomy. To accommodate the individual anatomy of the patients as closely as possible, four product families have been developed with three different medial curvatures and adapted CCD angles (Caput-Collum-Diaphyseal) (cf. Figure 1). The resulting close fit of the implant along the calcar ensures its rotational stability. This design feature makes it possible to maintain a relatively small cross-sectional area of the implant, which in turn enables at least partial intraoperative compensation of certain deformities, such as high antetorsion or retrotorsion. With 14 different sizes per family, the entire product range comprises 56 stems.

As with any new hip implant design or any medical device, it is essential to verify the long-term behaviour of the implant. In the context of hip stems, subsidence, stability, and stress shielding are of particular interest. Biomechanical studies have shown that primary implant stability must be within the critical threshold for osseointegration, which is achieved by limited micromovement and migration as well as high rotational stability [14]. Mid-term studies have shown promising results with stable and reliable integration for a Fitmore^®^ hip stem [15]. The long-term in vivo behaviour of the Fitmore^®^ stem has been described in one study with a focus on clinical outcome, subsidence, and bony changes [16]. But up to now, there is no publication of how patient-related factors such as age, gender, or individual anatomy, or surgical factors such as the surgical side, approach, or the initial varus/valgus alignment of the stem, may influence the long-term position of the stem. We hypothesised that all of the above factors could potentially correlate with a change in long-term coronal alignment and magnitude of subsidence.

This retrospective cohort study analyses the in vivo behaviour of the first 141 consecutively implanted Fitmore^®^ hip stems over a 10-year period. The study includes an analysis of subsidence, stability, and varus/valgus alignment, as well as an evaluation of the influence of patient-related, anatomical, and surgical factors on implant behaviour.

## 2. Patients and Methods

This retrospective, monocentric cohort study includes the first 141 patients who received a Fitmore^®^ Hip Stem between June 2007 and December 2008. The only inclusion criterion was the implantation of Fitmore^®^ Hip Stem, and there were no exclusion criteria. To avoid any selection bias, even patients with a previous operation of the affected hip joint, respectively proximal femur were included. The surgical hip dislocation was performed in 9 patients. In 2 patients, a femoral neck fracture was treated with a cannulated screw osteosynthesis. A total of 1 pertrochanteric fracture was fixed with a femoral nail. 3 patients had an acetabular osteotomy to treat developmental dysplasia of the hip, and 4 patients received a derotation osteotomy of the femur. All of the fractures and osteotomies have healed in an expected manner.

The operations were performed by two senior orthopaedic surgeons (HN and TW). All operations were performed in the lateral decubitus position. A modified anterolateral approach according to Röttinger [17] (in a minimally invasive approach), a stepped trochanteric osteotomy [18], and in one case a transgluteal approach were used.

Baseline characteristics of the patients (i.e., age and gender) and the implants used (i.e., stem size, family, head size, and initial varus/valgus angle of the stem) were collected at the time of surgery. Standard, conventional radiographic images (specifically, a low centred anterior-posterior pelvic radiograph) were taken and analysed at the following intervals: postoperatively, after 6 weeks, after 1 year, after 5 years, and after 10 years.

**Figure 2:**
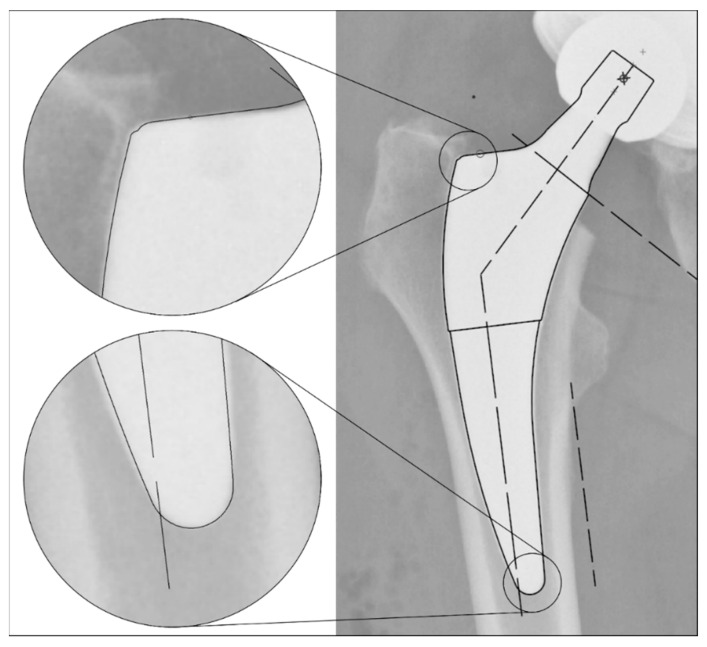
Method for calibrating the X-ray images: Equating the maximum length of the stem (from the shoulder to the tip) in the X-ray image to the template size known from the surgical report. This enables a comparable distance measurement even with different X-ray projections due to different rotation and/or varus/valgus position of the leg.

**Figure 3:**
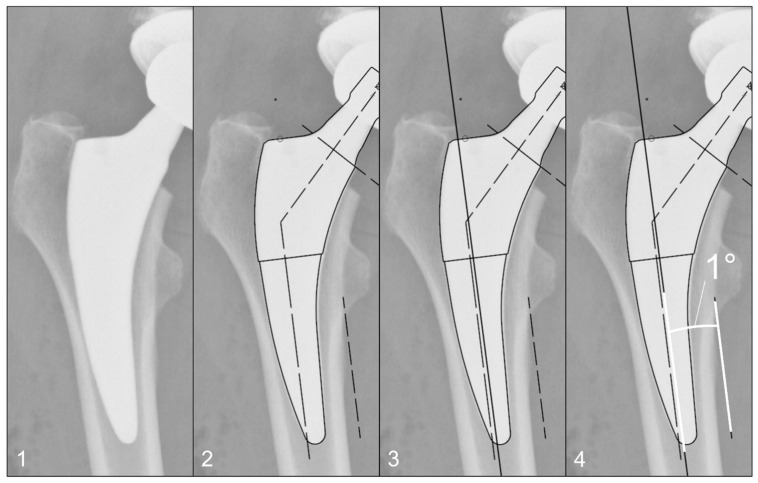
Method for measuring the varus/valgus angle of the stem: (**1**) Initial image. (**2**) Calibration of the image using a planning template, as shown in Figure 2. (**3**) Determination of the femoral longitudinal axis. (**4**) Measuring the angle between the femoral longitudinal axis and the long axis of the stem according to the planning template.

**Figure 4:**
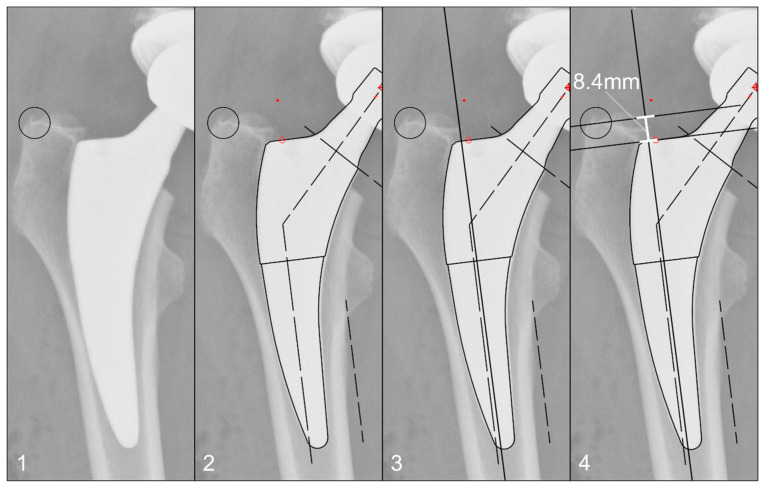
Method for measuring the stem position: (**1**) Identification of a clearly definable point on the greater trochanter, which appears identically on all radiographs up to 10 years postoperatively. (**2**) Calibration of the image using a planning template, as shown in Figure 2. (**3**) Determination of the femoral longitudinal axis. (**4**) Distance measurement of the defined point to the prosthetic shoulder along the femoral longitudinal axis.

The radiographs were analysed with TraumaCad^®^ software (Brainlab AG, Munich, Germany). We used only standardised deep-centered anteroposterior X-rays of the pelvis, with the central beam perpendicular to the midline and centered over the symphysis, and the legs positioned in 15° of internal rotation with the patient in the supine position. The images were calibrated by measuring the length of the depicted stem from its shoulder to its tip and equating it with the corresponding planning template. In this way, we could avoid any possible errors in our established calibration method with a reference sphere, which can be placed too far away from the central beam or too far away from the film. The measurements of the stem position were subsequently taken on the same axis to neutralise distortions caused by varying flexion and rotation positions of the leg during radiography (Figure 2). The varus/valgus alignment of the stems was determined by measuring the angle between the longitudinal axis of the stem and the longitudinal axis of the femoral canal (Figure 3). Subsidence was determined by measuring the distance from the shoulder of the stem to a clearly definable point at the greater trochanter in the previously determined longitudinal axis of the stem (Figure 4). Due to possible osseous changes over time, such as enthesiopathies or ossifications at the tip of the greater trochanter, defining this point was crucial to measure the correct distance.

Using the known stem and head size, as well as the initial varus–valgus angle, the true femoral offset was calculated, i.e., the distance from the centre of rotation to the longitudinal axis of the stem.

**Figure 5:**
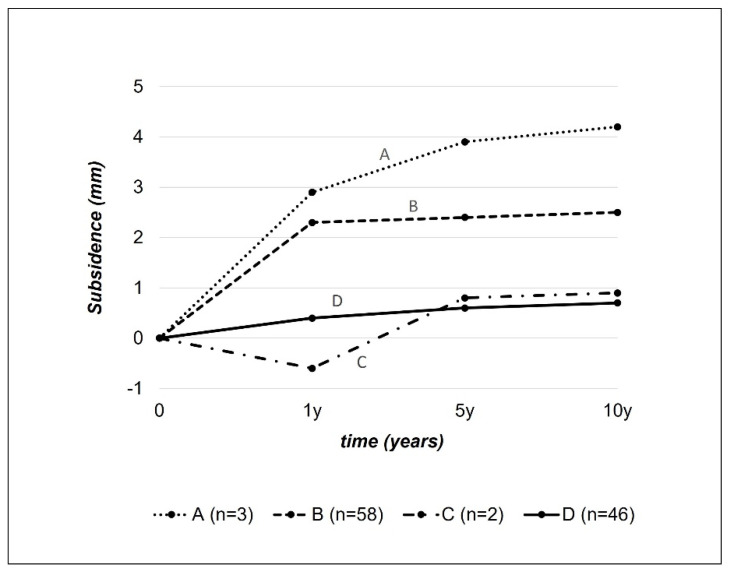
Migration patterns. Modification of the original description by Krismer et al. [19] according to our observation periods. Pattern A: subsidence > 1 mm both after 1 year and >1 mm between 1 and 10 years. Pattern B: subsidence > 1 mm after 1 year, then stabilisation. Pattern C: Stable situation after 1 year, then subsidence of >1 mm up to 10 years. Pattern D: Stable situation throughout the entire period up to 10 years without relevant subsidence.

Based on the measured subsidence over time, we assigned the stem behaviour to a modified migration pattern according to Krismer et al. taking into account the longer observation period of this study (cf. Figure 5) [19].

All radiographs were also assessed for radiological changes indicative of loosening (cortical hypo/hypertrophy, radioopacity) and categorised using an adaptation of the Gruen zones for short uncemented stems [20].

The statistical analysis of the data was carried out by the Institute of Mathematical Statistics at the University of Bern using SPSS^®^ Statistics (IBM, Armonk, NY, USA). The paired and triple interactions of the individual parameters were analysed using Bonferroni correction and the Brunner–Langer model for non-parametrical parameters. A significance level of *p* < 0.05 was used for the statistical analysis.

The study was approved by the Ethics Committee of the Canton of Bern, Switzerland. Prior to submission informed consent to publish the relevant information, including imaging was obtained from all patients.

## 3. Results

Of the 141 included patients, 118 completed the 10-year follow-up. Three patients died before concluding the follow-up, the other 20 were either lost on follow-up or refused to participate.

Due to the closure of the hospital where the operations took place, some radiographs were lost, resulting in 122 postoperative radiographs, 125 radiographs at the 6-week follow-up, 129 radiographs at the 1-year follow-up, and 131 radiographs at the 5-year follow-up. One stem had to be replaced during the observation period. The stem was judged to be too small on the post-operative radiograph and was therefore replaced with a larger stem three days after the initial surgery. The case was followed up as planned.

Analysis of the varus/valgus alignment showed on average a progression into varus within the first 5 years. There was no further change after that. The change in angulation was only statistically significant comparing the different time points with the 6 weeks status. In between all other time points the changes in varus/valgus alignment were not statistically significant (cf. Table 1). At six weeks, 57.1% of total cumulative angle change was seen, and at one year, 85.7%. 

Stems implanted in a valgus alignment (i.e., initial postoperative angle <0°; n = 13, 9.8%) showed a mean progression of +1.5° over 10 years, those with a neutral alignment (i.e., 0–3° of initial postoperative varus alignment; n = 73, 51.8%) of +0.7° over ten years, while those with a primary varus alignment (i.e., >3° of initial postoperative varus alignment; n = 55, 39%) showed a mean progression of +0.5°.

The average angular progression after ten years in patients with a small postoperative total offset of <41 mm (n = 47, 33.3%) was +0.8°, in those patients with a medium total offset of 41–46 mm (n = 48, 34%) as well as in patients with a high total offset of >46 mm (n = 46, 32.6%) the mean progression was +0.7°.

Within the different stem families, there was an average angular progression over ten years from 0.5° to 1.5° in A-stems, 2.2° to 2.8° in B-stems, 3.2° to 4° in extended B-stems, and 4.6° to 5.3° in C-stems.

Regarding the relation of stem size and angular progression, stem sizes of 3 or smaller (n = 29, 20.6%) showed an average change of +1.1° after 10 years, stems of size 4–6 (n = 69, 48.9%) of +0.7° and stem size 7 or larger (n = 43, 30.5%) of +0.6°.

Right hips (n = 74, 52.5%) had a total angular progression of +0.7° and left hips (n = 67, 47.5%) of +0.8° after 10 years.

After the anterolateral approach (n = 125, 88.7%) the varus/valgus alignment changed on average of +0.7° and after trochanteric osteotomy (n = 15, 10.6%) of +1.1°. Due to the singular occurrence of a lateral transgluteal approach, no evaluation was possible for this case (cf. Table 2).

The hip stems in men tended to be implanted more varus than in women. The average initial postoperative varus angle in men was 4°, compared to 1.7° in women. However, the comparison between male and female total varus progression over ten years of 0.7° and 0.8°, respectively was not statistically significant.

Younger patients tended to have a greater increase in varus angulation over 10 years. In younger patients (<45 years at the time of surgery; n = 25, 17.7%), there was an increase in the varus angle of +1.0°, in patients aged between 45–70 years (n = 90, 63.8%) of +0.7° and in older patients (>70 years; n = 26, 18.4%) of +0.6°.

But, none of the examined factors (initial postoperative varus/valgus alignment, postoperative total offset, stem family, stem size, surgical side, approach, gender, nor age) had a statistically significant influence on varus/valgus alignment.

**Figure 6:**
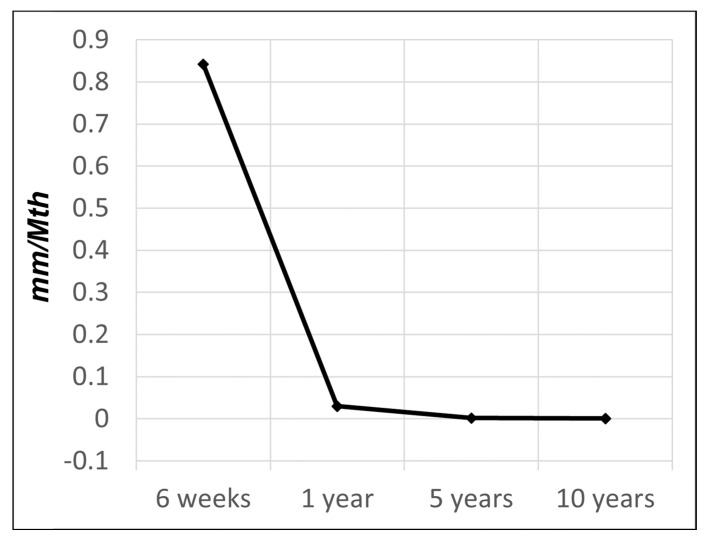
Rate of subsidence over time: 0.842 mm/Mth up to 6 weeks, then 0.030 mm/Mth up to 1 year, 0.002 mm/Mth up to 5 years, and 0.001 mm/Mth up to 10 years.

Analysis of the subsidence showed on average a progression over the entire observation period of 10 years. The subsidence was statistically significant between consecutive time points up to 5 years. But, there was no statistical significance of subsidence between years 5 and 10 (cf. Table 2). However, the subsidence rate decelerated significantly over time and was 0.842 mm/Mth in the first 6 weeks, 0.030 mm/Mth from week 6 to 1 year postoperatively, 0.002 mm/Mth between 1 and 5 years, and 0.001 mm/Mth between 5 and 10 years (cf. Figure 6). A total of 70% of the total subsidence took place within the first 6 weeks.

There were no statistically significant differences between the two genders (1.8 mm for men after 10 years vs. 1.7 mm for women after 10 years) nor was there a statistically significant difference among the age groups. Both the younger (<45 years) and the older patients (>70 years) had subsided on average of 2.0 mm, and the group between 45–70 years subsided on average 1.5 mm.

The initially in a varus position implanted stems subsided slightly more (1.8 mm after 10 years) than the valgus implanted ones (1.2 mm after 10 years).

There were no significant differences between the stem families. The A stems tended to subside slightly less (1.1 mm after 10 years). There were also no significant differences in the total offset and stem sizes.

With 1.8 mm in 10 years, stems with an anterolateral approach subsided significantly (*p* = 0.0222) more than those with a trochanteric osteotomy (0.9 mm after 10 years).

The analysis of the migration patterns, based on the modified classification of Krismer et al. showed pattern A in 1.8% of patients, pattern B in 53.2% of patients, pattern C in 2.8% of patients, and pattern D in 42.2% of patients. 

At 10 years of follow-up, there was no conventional radiological evidence of stem loosening, periprosthetic fracture, or dislocation in any of the hip stems.

## 4. Discussion

Overall, there were only minor changes in the varus/valgus alignment and subsidence. On average we observed a varus progression of +0.7° and a subsidence of 1.7 mm over 10 years. Most of the changes in angulation and subsidence were observed in the first six weeks after surgery. Thereafter and especially after 1 year, the changes in both parameters were hardly measurable. This finding is compatible with the assumption that small changes in position are still possible as part of the osteointegration of the implant through bone in- and on-growth onto the rough surface of the stem [21,22,23,24]. However, once this integration is complete, the stem remains stably in place over the years. This observation has already been made on a mid-term basis for the Fitmore^®^ Hip Stem [15,25]. A recently published study on the 10-year results of the Fitmore^®^ Hip Stem showed an increase in subsidence from 1.6 to 5 mm from the first to the tenth year, which is somewhat contradictory to our results [16]. In our study population, subsidence had largely stopped after 1 year, as also observed by Freitag et al. 2 years after implantation [15]. The divergent results are difficult to reconcile for now and certainly require further observation.

Recent studies have shown good results regarding long-term stability in other uncemented hip stems as well. Kutzner et al. measured an average subsidence of 1.43 mm after 2 years in the Optimis^®^ short stem (Mathys Medical AG®, Bettlach, Switherland), indicating a subsidence below the threshold of 1.5 mm according to Krismers et al. [19,26]. Further results from this cohort are still lacking. In the cohort of von Lewinski et al., almost 2000 cases of the Metha^®^ short stem (Aesculap® AG, Tuttlingen, Germany) were observed for 10 years. Overall, 1.9% of implants needed stem revision, and only 0.5% of them due to major stem subsidence. The authors suggested stem undersizing as the main reason (58%) for mechanical problems leading to revision [27]. In a small study of 26 patients, Sesselman et al. found a mean subsidence after ten years of 0.22 mm in the Cerafit^®^ short stem (Ceraver®, Roissy-en-France, France) [28]. And, in the study of Yamasaki et al., all of the 194 implanted CLS Spotorno^®^ stems (Zimmer Biomet, Warsaw, IN, USA) subsided less than 2 mm, demonstrating its long-term stability even after 10 years [29]. Interestingly, longer-term results are still lacking, even though many of these cementless short-stem designs have now been routinely implanted for almost 2 decades.

As mentioned above, the Fitmore^®^ hip stem is inserted along the anatomic pathway with a close fit to the calcar in the final position, which provides its rotational stability. Accordingly, one would expect that subsidence takes place along this medial curvature leading to a valgus tendency. The results now clearly show that the stems tend to angulate into varus. At the same time, our results show a tendency towards more pronounced changes the more valgus alignment is present right after implantation. The subsequent angle changes follow the lever arm until the implant is in stable contact with the medial calcar with corresponding bony resistance, which prevents further relevant angle changes after a few weeks/months postoperatively. We attribute the marked angular changes in the first 6 weeks to the fact that, particularly in osteoporotic bone, high loads on the calcar are avoided during implantation to prevent fractures, so that optimum contact with the calcar is achieved through loading. Accordingly, we saw no correlation between the angular change (on average into varus) and the subsidence of the stems. The even lower tendency to angular change after trochanteric osteotomy indicates that the fixation of the prosthesis around the prosthetic shoulder is of minor importance, but that the contact at the calcar and the metaphyseal cortex is crucial for stable fixation. 

Our data show no significant changes in angular positioning after the first postoperative year. The situation is similar for subsidence, with statistically significant changes observed up to the 5th postoperative year. However, the additional 0.2 mm of subsidence between years 1 and 5 is unlikely to be clinically significant. In fact, we believe that the observed differences fall below the threshold of measurement accuracy of our method and, as such, do not consider them to be meaningful. The absence of significant changes after 1 and 5 years, respectively suggests that long-term stability and integration can be assumed after a period of initial settling. These findings are consistent with previous studies with shorter observation periods [15]. The extended observation period in this study confirms that the position reached after 1 year is indeed the final position of the stem. For this reason, a successful and long-lasting osteointegration of the implant can be assumed.

A total of 4.6% of stems showed an unfavourable migration pattern (Krismer [19] patterns A and C) without a single case of stem loosening after ten years. The question therefore arises as to whether the unfavourable migration patterns apply equally to short-curved and long-straight stems or whether other as yet unidentified factors are of greater significance. An even longer observation period may be required.

Neither the patient factors (gender, patient age), the surgical factors (primary implantation angle, surgical approach, surgical side) nor the implant factors (stem family, size, total offset) showed a significant influence on the angular alignment or the subsidence of this short uncemented hip stem which is even more remarkable as there were no patient selection criteria for the implantation.

This study is limited by preoperative selection bias due to surgeon implant choice, which may affect the generalisability of the results. In addition, the lack of preoperative radiographs for bone morphology classification prevents assessment of the impact of bone structure on surgical outcomes. Due to the long study period, some of the ten-year results are missing. These patients either died, could not be contacted, or refused to participate. 

Nevertheless, a 100% survival rate after 10 years (except for the single case of early postoperative stem exchange after 3 days due to undersizing in the postoperative X-ray control) is remarkable.

## 5. Conclusions

The results of this 10-year retrospective cohort study show that changes in stem position with respect to varus/valgus alignment occur mainly in the first 6 weeks and that the stem has found its position by one year at the latest and remains stable thereafter. Subsidence generally concludes within one year and is definitively complete by no later than five years. The favourable biomechanical behaviour is reflected in the register data, which is as good as or better than that of established uncemented long stems [30].

Furthermore, the long-term behaviour of the stem is not significantly influenced by patient demographics, surgical technique, or implant-specific factors, demonstrating its versatility and reliability in a wide range of indications.

However, more long-term studies of implant behaviour in uncemented hip stems are needed to fully understand implant behaviour and the factors that influence it, such as the effect of different preoperative bone qualities.

## Figures and Tables

**Table 1:** Basic Characteristics of Patients, Surgical Procedures and Implants at Baseline.

Patient Characteristics		Population Total (n = 141)
Age at surgery (years)	total (SD)	57.4 (±12.7)
Gender	male	63 (44.7%)
	female	78 (55.3%)
Primary Diagnosis	Primary OA	97 (68.3%)
	OA in acetabular dysplasia	16 (11.3%)
	OA in femoroacetabular impingement	8 (5.6%)
	OA in femoral head necrosis	7 (4.9%)
	other specified conditions	13 (9.1%)
**Surgical Procedure Details**		
Surgical Approach	Modified anterolateral	125 (88.7%)
	Stepped trochanteric osteotomy	15 (10.6%)
	Lateral transgluteal	1 (1.7%)
Surgical Side	right	74 (52.5%)
	left	67 (47.5%)
**Implant Details**		
Stem Family	A	17 (12.1%)
	B	65 (46.1%)
	B extended	44 (31.2%)
	C	15 (10.6%)
Total postoperative offset	total	43.9 (±6.8)
	male	48.1 (±6.4)
	female	40.5 (±5.0)
Initial Varus/Valgus Alignment	total (range)	2.8° (−6°–+11°)
(Varus > 0°)	male (range)	4.0° (−6°–+11°)
	female (range)	1.7 (−3°–+8°)

**Table 2:** Varus/Valgus Angulation and Subsidence over Time.

	Postoperative	6 Weeks	1 Year	5 Years	10 Years
**Average Varus/Valgus Angulation (°)**	2.8 (±2.7)	3.2 (±2.7)	3.4 (±2.7)	3.5 (±2.7)	3.5 (±2.6)
		*p* < 0.0001			
		*p* < 0.0001			
		*p* = 0.0002			
**Average Subsidence (mm)**	n/a	1.2 (±1.2)	1.4 (±1.4)	1.6 (±1.4)	1.7 (±1.4)
		*p* < 0.0001			
			*p* < 0.0001		
				*p* < 0.1697	

## Data Availability

The data supporting the findings of this study are available from the corresponding author upon reasonable request. Due to legal limitations, access to the data may be provided under certain conditions.
